# Influence of age on postural sway during different dual-task conditions

**DOI:** 10.3389/fnagi.2014.00271

**Published:** 2014-10-22

**Authors:** Marco Bergamin, Stefano Gobbo, Tobia Zanotto, John C. Sieverdes, Cristine L. Alberton, Marco Zaccaria, Andrea Ermolao

**Affiliations:** ^1^Sport and Exercise Medicine Division, Department of Medicine, University of PadovaPadova, Italy; ^2^College of Nursing, Medical University of South CarolinaCharleston, SC, USA; ^3^Physical Education School, Federal University of PelotasPelotas, Brazil

**Keywords:** dual-task performance, aging, stabilometry, assessment methods, postural sway

## Abstract

Dual-task performance assessments of competing parallel tasks and postural outcomes are growing in importance for geriatricians, as it is associated with predicting fall risk in older adults. This study aims to evaluate the postural stability during different dual-task conditions including visual (SMBT), verbal (CBAT) and cognitive (MAT) tasks in comparison with the standard Romberg's open eyes position (OE). Furthermore, these conditions were investigated in a sample of young adults and a group of older healthy subjects to examine a potential interaction between type of secondary task and age status. To compare these groups across the four conditions, a within-between mixed model ANOVA was applied. Thus, a stabilometric platform has been used to measure center of pressure velocity (CoPV), sway area (SA), antero-posterior (AP) and medio-lateral (ML) oscillations as extents of postural sway. Tests of within-subjects effects indicated that different four conditions influenced the static balance for CoPV (*p* < 0.001), SA (*p* < 0.001). *Post-hoc* analyses indicated that CBAT task induced the worst balance condition on CoPV and resulted in significantly worse scores than OE (−11.4%; *p* < 0.05), SMBT (−17.8%; *p* < 0.01) and MAT (−17.8%; *p* < 0.01) conditions; the largest SA was found in OE, and it was statistically larger than SMBT (−27.0%; *p* < 0.01) and MAT (−23.1%; *p* < 0.01). The between-subjects analysis indicated a general lower balance control in the group of elderly subjects (CoPV *p* < 0.001, SA *p* < 0.002), while, the mixed model ANOVA did not detect any interaction effect between types of secondary task and groups in any parameters (CoPV *p* = 0.154, SA *p* = 0.125). Postural sway during dual-task assessments was also found to decrease with advancing age, however, no interactions between aging and types of secondary tasks were found. Overall, these results indicated that the secondary task which most influenced the length of sway path, as measured by postural stability was a simple verbal assignment.

## Introduction

Elderly subjects show a decline in the performance during a combination of motor and cognitive tasks (Boisgontier et al., 2013). A key-role is played by the structural neurodegenerative and neurochemical changes occurring as part of the normal aging process; that results in less efficient integration of visuo-spatial and sensorimotor processing affecting changes in the way the motor system is activated (Ward and Frackowiak, 2003). Hence, increased cognitive demands are required to determine adaptive compensation processes (Seidler et al., 2010) to sustain performance.

Several theories have been postulated to explain the contribution of cognitive loads that influence the concurrent motor control, or the global performance. Of these, some were focused on the information processing bottleneck (Borst et al., 2010), others on interference (Wickens, 2008) and attention resources (Verhaeghen et al., 2003), and more recently, one that includes a structure-function-behavior model to explain the influence on postural control (Papegaaij et al., 2014).

In the clinical setting, dual-task performance is of growing importance for geriatricians when the motor task encompasses postural balance on upright position, since its impairment is unavoidably associated with increased fall risk (Muir et al., 2010). Exercise, in general, and specific balance-target interventions have been shown to improve postural sway (Cadore et al., 2013), however, it seems that exercise is not as beneficial in improving static balance during dual-task situations in healthy elderly (Gobbo et al., 2014), while, oppositely, older adults with neurological impairment can be benefited (Zanotto et al., 2014). Recent reviews (Gobbo et al., 2014; Zanotto et al., 2014), together with opinions of Agmon et al. (2014) have agreed about the lack of a simple and easy-administrable method to measure the postural stability during dual-task conditions, that concretely impedes an accurate comparison among the studies. Moreover, a standardized measurement method is also needed because the performance can also be modified by focusing attention on one task or another (Kramer et al., 1995) and for the biases in the postural data acquisition, which are highly alterable to manual responses and visual fixation (Stoffregen et al., 1999). Maintaining an upright stance under conditions that challenge balance, is often found to affect concurrent cognitive task. On the other hand, the ways in which cognitive tasks impact postural performance are less reliable (Fraizer and Mitra, 2008). From this point, if the main information needed is not the dual-task interference, but the balance performance during dual-tasking, a standardized cognitive task could be more effective to analyze postural sway variations, as previously highlighted for dual-task assessment that included walking (Al-Yahya et al., 2011; Chu et al., 2013).

It is important to identify not only how dual tasks effects which static balance performance measures in elderly, but to conceptualize how the progression of measurements change through the lifespan. Therefore, this study sought to investigate the postural sway over various dual task conditions in younger and older individuals.

## Materials and methods

### Participants

Thirty young adults (15 men and 15 women, aged from 18 to 28) and 30 older adults (15 men and 15 women, age > 64) has be recruited from public announcement visible on the notice board in the Sport and Exercise Medicine Division, Department of Medicine, University of Padova (Padova, Italy). Eligibility criteria were implicated so as to not include health problems or physical limitations that could affect the study results. Potential subjects who responded to the announcements were screened by research staff using inclusion and exclusion criteria and completed an informed consent process. Upon consent, an anamnesis was then performed by a physician. Exclusion criteria included: uncorrected visual impairment, neurologic pathology (e.g., Parkinson's disease, stroke), orthopedic surgery to the lower limbs, medication that could influence posture and/or gait, neurological disorders, cognitive impairments and a history of vertigo or falls in the previous month.

The study complied with the current laws of Italy for research on human participants and was approved by the local review board.

### Procedure

Before testing, a medical history questionnaire was administered together with the Mini-Mental State Examination (Folstein et al., 1975), used as a screening device for cognitive impairments (Tombaugh and McIntyre, 1992); cut-off value was 26 points. Height was measured to the nearest cm using a stadiometer (Ayrton Corporation, Model S100, Prior Lake, MN). Weight was measured using an electronic scale (Home Health Care Digital Scale, Model MC-660, C-7300 v1.1) to the nearest 0.1 kg. Descriptive variables are presented in Table 1.

**Table 1:** **Characteristics of the sample**.

	**Young subjects (*n* = 30)**	**Elderly subjects (*n* = 30)**
Age (years)	23.11±1.58^*^	71.92±5.74^*^
Height (cm)	173.73±9.25^*^	164.4±8.35^*^
Weight (kg)	68.36±11.49	69.03±13.24
BMI (kg/m^2^)	22.50±2.06^*^	25.38±3.63^*^
Foot number (cm)	40.93±2.79	39.53±2.71
MMSE (score)	29.23±0.76	28.16±1.34

*This table describes the characteristics of participants*.

* *Indicates a statistically significant difference between the two groups. BMI, body mass index; MMSE, mini mental state examination*.

The ARGO stabilometric platform (RGMD, Genova, IT) has been used to measure the center of pressure (CoP) coordinates in the anterior-posterior and medial-lateral directions (Scoppa et al., 2013). These oscillations were collected at 100 Hz sampling rate; we filtered raw data using the ARGO software (RGDM, Genova, IT) that adopts a post-processing low-pass filtering with a 10 Hz frequency cutoff. The normalization of CoP velocity (CoPV) and sway area (SA) data is set by default from the ARGO software, by dividing the CoPV and SA measures by the duration of the test. CoPV was calculated by taking the coordinates of two consecutive points and calculating the distance between the two by using the Pythagorian theory and adding all the distances together (mm/s.) (Dault et al., 2003). SA describes the enclosed area covered by the CoP as it oscillated within the base of support (mm^2^/s) (Kim et al., 2009) along the antero–posterior (AP) and medio–lateral (ML) axes. AP and ML sways expresses the range of CoP displacements in the sagittal and frontal planes, respectively.

The stabilometric platform is fixed to the floor, 3 m away from walls or objects that could be used as a support. The platform was positioned 3 m from a whiteboard, which was used during the test as a focal point for the subject.

#### Single task (OE)

Single task balance measures consisted of the Romberg test. In this test, the investigator instructed the subjects to stand upright on the stabilometric platform with feet together (Lanska and Goetz, 2000) and keep they eyes open for 30 s.

#### Dual-task

A vocal registration explained each cognitive task before the beginning of each trial. The specific duration of each secondary task was 30 s.

***Spatial-memory brooks test (SMBT)***. Spatial-Memory Brooks test was used as one of the secondary tasks. Following the methods used by Swan et al. (2004), subjects listened to a series of sentences through computerized audio tracks while performing the primary task. For each trial, “4 per 4” matrices were described with a series of eight sentences. The first sentence was always, “*In the starting square insert 1*.” Each matrix had the same “*starting square*.” The number 1 was located in the square, in the second row from the top and on second column from the left. Subsequent sentences instructed the subjects to place the next number in a square to the right, left, up, or down from the previously filled square; for example, “*In the next square to the left insert 2*, *in the next square down put a 3*, (…).” There were 2 practice run-in trials before the study measurement were taken. The numbers 2 through 8 were “placed” so that a number was always located directly above or below, or to the right or the left of the number preceding it. Numbers never fell outside the matrix or in the same square as another number and always ordered from 1 to 8 (Kerr et al., 1985).

***Counting backwards aloud test (CBAT)***. In the counting backwards condition, participants were provided a starting number, randomly selected from 90 to 100, and were asked to count backwards aloud as fast and as accurately as possible for the duration of the trial (Yardley et al., 1999). “The specific instruction was: ‘*Count backwards from the number 93*.”’

***Mental arithmetic task (MAT)***. In this condition, participants were asked to wordlessly solve simple addition and subtraction mathematical problems and to verbalize the response when requested by the experimenter at the end of the trial. A computerized audio recording was used to administer the arithmetic problem requiring additions and subtractions of a series of single digit numbers (Vuillerme and Vincent, 2006), for example “*add 4 (pause); subtract 3 (pause); add 5* (…).”

The order of experimental conditions (single task, dual tasks) was randomized while each test was repeated twice. The sessions were separated by 5 min of rest to reduce potential fatigue effects.

Along with the postural sway data, cognitive task performances have also been calculated for each individual. About spatial-memory Brooks test (SMBT), scores represents the average correct replies where the maximal individual score was 7. About counting backward aloud test (CBAT) and mental arithmetic task (MAT) errors were reported as sum for each group for each trial. The average error scores for the two groups are reported in the Supplementary Table 1.

### Statistics

#### Sample size calculation

Sample size calculation (*N*) was based on the mean values of CoPV detected from past studies (Kerr et al., 1985; Yardley et al., 1999). Sample size was calculated with the following equation for each condition:

$$N=\frac{2SD^{2}{\left(Z_{\alpha }+Z_{\beta }\right)}^{2}}{{△}^{2}}$$

*SD* is the standard deviation detected in a previous study based on the mean value of the CoPV; *Z*_α_ is represented by α = 0.05 (1.96) and meaning the α point at which a null hypothesis is rejected, 1-*Z*_β_ represents the 80% existing chance that a false null hypothesis is rejected; Δ is the mean meaningful difference to determine a improvement in CoPV.

The equation estimated a sample size of 24 that would reflect a result with 80% power. To ensure an adequate sample size, we oversampled at 30 to allow for a 20% dropout rate. The final number of participants recruited numbered 60 subjects (30 young and 30 elderly subjects).

#### Statistical analysis

Statistical analysis was performed using SPSS (Version 18.0 for Windows, SPSS Inc., Chicago, IL). Demographic results were expressed as means ± standard deviation (SD) or as percentages. The Kolmogorov-Smirnov (K-S) demonstrated a normal distribution of the data. Mann-Withney *U*-test was applied to compare scores for secondary tasks, while, to examine the interaction between the different tasks (OE, SMBT, CBAT, and MAT) and the groups (young vs. elderly) on static balance outcomes, a 2 × 4 between-within model ANOVA was applied. Fisher's least significant difference *post-hoc* contrasts (in the within model) with Bonferroni correction were applied. Significance limits were set at alpha level of *p* = 0.05.

## Results

Considering the whole sample, tests of within-subjects effects indicated that different conditions (OE, SMBT, CBAT, and MAT) influenced all the outcome variables: CoPV (*F* = 20.083, *p* < 0.001), SA (*F* = 14.838, *p* < 0.001), AP (*F* = 15.469, *p* < 0.001), and ML (*F* = 4.586, *p* = 0.006).

*Post-hoc* analyses of the whole sample are reported on Table 2. Mean differences reported in Table 2 indicated that CBAT task induced the worst condition on CoPV (15.89 ± 6.21 mm/s, mean ± *SD*), in fact CoPV resulted significantly higher than during OE (−11.43% with respect to CBAT), SMBT (−17.77%), and MAT (−17.80%) conditions. The largest SA was found in OE condition (29.15 ± 16.14 mm^2^/s, mean ± *SD*), and it was statistically larger than dual-tasking with concurrent SMBT (−26.97% with respect to OE) and MAT (−23.13%). There was not a statistically significant difference between OE and CBAT conditions. Single task condition (OE) determined the largest oscillation on AP sway measures (23.29 ± 7.41 mm, mean ± *SD*), with *post-hoc* contrasts that showed statistically significant differences when comparing OE vs. SMBT (−21.91%), vs. CBAT (−10.60%), and vs. MAT (−15.31%). ML sway measures for the OE condition had the largest sway (23.68 ± 7.78 mm, mean ± *SD*), followed by CBAT (20.29 ± 7.41 mm, mean ± *SD*). No differences between these were found, however, statistical significant differences were found between OE vs. SMBT (−10.51%) and OE vs. MAT (−10.34%).

**Table 2:** ***Post-hoc* contrasts on center of pressure measures during single task and during different secondary conditions**.

		**Mean difference on CoPV (mm/s)**	**Mean difference on SA (mm^2^/s)**	**Mean difference on AP sway (mm)**	**Mean difference on ML Sway (mm)**
OE	SMBT	1,0067	7,8642^**^	5,1044^**^	2,4888^*^
	CBAT	−1,8172^*^	1,6276	2,4685^*^	2,0084
	MAT	1,0121	6,7439^**^	3,5665^**^	2,4488^*^
SMBT	OE	−1,0067	−7,8642^**^	−5,1044^**^	−2,4888^*^
	CBAT	−2,8239^**^	−6,2366^*^	−2,6358^*^	−0,4804
	MAT	0,0054	−1,1203	−1,5379	−0,0400
CBAT	OE	1,8172^*^	−1,6276	−2,4685^*^	−2,0084
	SMBT	2,8239^**^	6,2366^*^	2,6358^*^	0,4804
	MAT	2,8293^**^	5,1163^*^	1,0979	0,4404
MAT	OE	−1,0121	−6,7439^**^	−3,5665^**^	−2,4488^*^
	SMBT	−0,0054	1,1203	1,5379	0,0400
	CBAT	−2,8293^**^	−5,1163^*^	−1,0979	−0,4404

*This table shows the post-hoc comparisons with the mean differences on CoPV, center of pressure velocity; SA, sway area; AP, antero-posterior; ML, medio-lateral, sways across the different secondary conditions: Romberg's OE, open eyes standard position; SMBT, spatial-memory Brooks test; CBAT, counting backward aloud test; MAT, Mental arithmetic task*.

* p < 0.05 and

** *p < 0.01 indicate a statistically significant difference in the pairwise comparison, mm, millimeter; s, second*.

**Figure 1:**
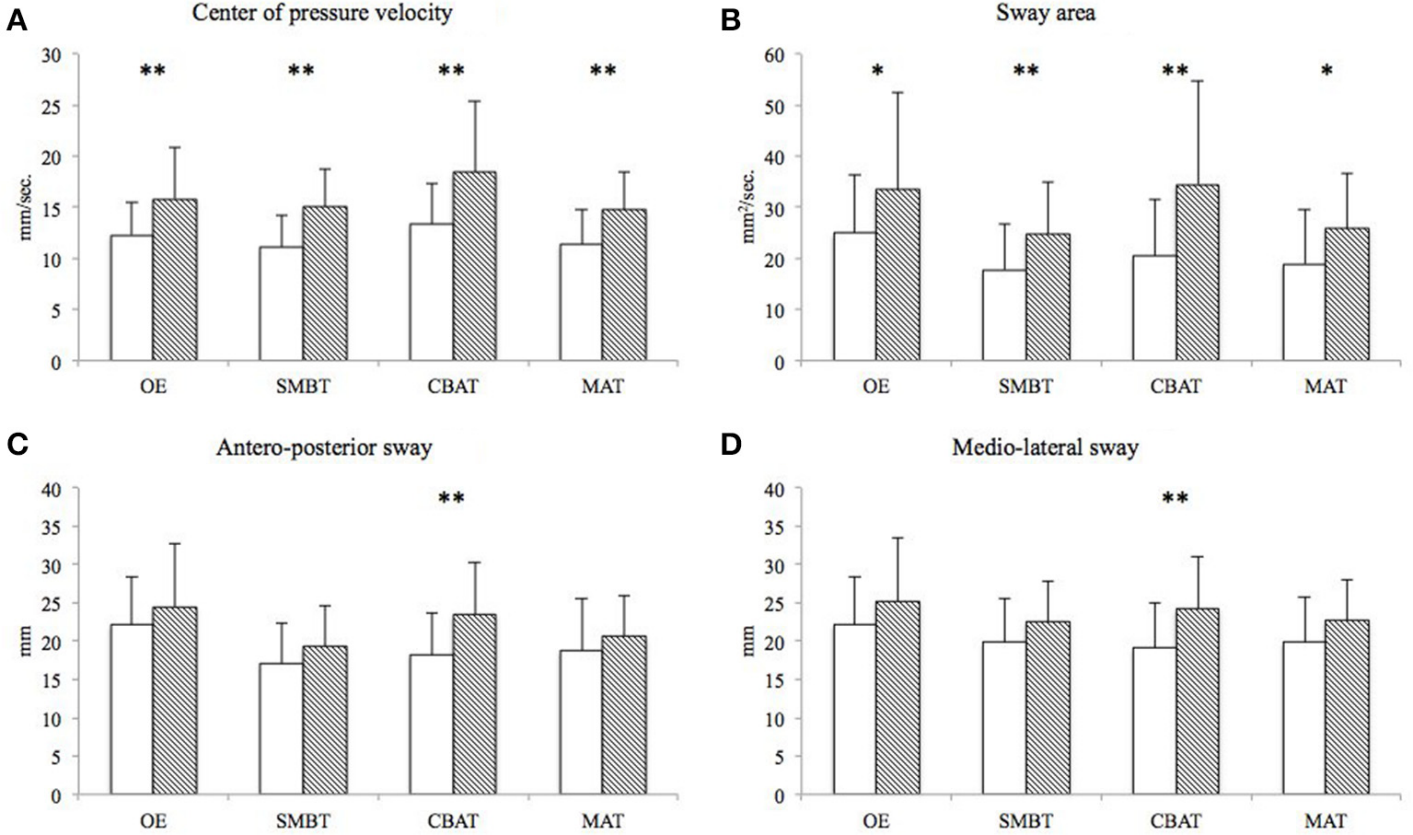
**White columns represent the mean (±SD) values of young subjects on the CoPV (A), SA (B), AP (C), and ML sways (D)**. Shaded columns represent values for the elderly participants. ^*^p < 0.05 and ^**^p < 0.01 indicate a statistically significant difference between young and elderly participants. CoPV, center of pressure velocity; SA, sway area; AP, antero-posterior sway; ML sways, medio-lateral sway; mm, millimeter; s, second.

The between-subjects analysis indicated a general lower balance control in the group of elderly subjects (CoPV, *F* = 15.412, *p* < 0.001; SA, *F* = 10.768, *p* < 0.002; AP, *F* = 4.961, *p* = 0.03; ML, *F* = 6.464, *p* = 0.014). Both CoPV and sway area values were lower in young subjects and in all conditions (see mean values ± SD on Figures 1A,B). Finally, for AP and ML sways, a significant difference between the two groups was observed (see mean values ± SD on Figures 1C,D).

Mixed modeling ANOVAs did not detect any interaction effect between type of task and groups for all balance parameters (CoPV, *F* = 1.774, *p* = 0.154; SA, *F* = 1.941, *p* = 0.125; AP, *F* = 1.937, *p* = 0.125; ML, *F* = 1.255, *p* = 0.292). Although elderly subjects showed a trend for reduced dual-task ability with CBAT across all test conditions, there were no indication of relationship between the groups and the dual-task performance during different tasks.

## Discussion

This investigation aimed to compare the responses of different secondary tasks during dual-task conditions on static balance in young adults and healthy elderly subjects. These secondary tasks included solving spatial-memory Brooks test, counting aloud backwards and answering arithmetic equations. The main result of this study implied that a simple verbal assignment was the secondary task which largely increased the velocity of the CoP, seemingly indicating a higher request of postural adaptation; this phenomenon appeared in the whole sample as was not sensitive to age status. Furthermore, together with standard Romberg's OE position, CBAT was the condition that determined a largest SA. On the other hand, SMBT and MAT conditions showed a better performance on CoPV, SA, antero-posterior and medio-lateral sways, confirming that a secondary task not necessarily negatively affect this kind of measures (Maylor et al., 2001).

This protocol also highlighted that postural sway increases with advancing of age, however, within the conditions compared in this study, we did not find an interaction between age and type of secondary task. In other words, comparing younger subjects with the elderly counterparts, the magnitude of difference among OE, CBAT, MAT, and SMBT was not so sufficiently large to determine a statically significant modification for all the postural stability parameters.

Our results are aligned with those reported by Yardley et al. (1999) which showed an increase in the length of sway during a spoken mental arithmetic task (e.g., counting backwards by multiples of seven). Increasing respiratory frequency generates an increase of the center of pressure length (Hodges et al., 2002). Consequently, higher perturbations on postural sway during verbal secondary task can probably be ascribed to the respiratory muscle activity in relation to vocalization. From another standpoint, Ceyte et al. (2014) found that counting backwards (e.g., subtracting by 3 or 13 digits), as secondary task, affected static balance, suggesting that the increase in sway length may be related to the use of oculomotor activity as unintentional attempts to increase arousal by self-generated body movement. Nevertheless, during verbal assignment there is also cognitive activation; for instance, a simple monolog (e.g., describing a familiar place) determined such a significant effect of cognitive load to modify postural stability (Holmes et al., 2010). Thus, it is still unclear if it is the vocalization *per se*, the cognitive task needed to count backwards, or both together, which are responsible for the increase of CoPV and SA.

Dual-tasking with SMBT and MAT conditions globally showed better performances on CoPV, SA, antero-posterior, and medio-lateral sways. Our results are in agreement with data reported by Swan et al. (2004) and Vuillerme and Vincent (2006), which respectively observed improvements in postural stability adding a visuo-spatial assignment and a mental arithmetic calculation during secondary tasks. Adding a secondary cognitive task produces better balance performance than Romberg alone (i.e., performing only the primary task), in contrast with classic theories. These results probably can be explained in these terms: when a subject concentrated only on postural tasks, they swayed more because they focused into itself. In contrast, an external focus condition, compared to an internal focus condition promotes greater automaticity in movement control (Cluff et al., 2010). In addition, a concurrent secondary cognitive task could tense postural muscles, which may result in the adoption of a stiffening strategy to regain posture (Carpenter et al., 2001). Furthermore, diverting attention from the control of posture could result in the loss of small exploratory movements of the feet (Vuillerme and Vincent, 2006). On the contrary, the attention demands of balance control vary depending on the complexity of the task and the type of secondary task being performed (Woollacott and Shumway-Cook, 2002).

Examining the aging effects on this study's sway measures, all conditions determined a higher CoPV and SA for the elderly group when compared to in the young sample. A further finding was also the significant difference between the two groups in AP and ML sways during the CBAT condition (Figures 1C,D). These data may suggest a greater instability, for the elderly, during the CBAT condition. However, while the CoPV and SA differences seem to be the consequence of an increase in those values with respect to the OE condition (Romberg's standard positioning), the significant differences detected on AP and ML sways appear more the consequence of a reduction of these oscillations in the younger group. These results are consistent with papers reporting reduced postural sway when young subjects engage in concurrent motor and cognitive tasks (Balasubramaniam and Wing, 2002; Yeh et al., 2014) or divert their attention from postural control (McNevin and Wulf, 2002). One potential mechanism contributing to the reduction on postural sway during dual-task conditions is the increase on baseline muscle activity (Loeb et al., 1999). Indeed, in these conditions, the observed muscle co-activation at the ankle joint could produce an increase of reflexive muscle activity and explain the reduction on postural sway (Dault et al., 2001; Ehrenfried et al., 2003; Weeks et al., 2003). On the other hand, standing is only an apparently automatic motor task, which, in the elderly, may require further cognitive resources, due to the reduction in sensorimotor and cognitive attention functions (McDowd, 1997; Wade and Jones, 1997; Lacour et al., 2008; Yeh et al., 2014). This may contrast the reduction in postural sway (observed on AP and ML sways in the younger group) when elderly subjects engage in cognitive or motor tasks during standing balance.

As mentioned, the mixed-model analysis did not show any interaction between the types of secondary task and the groups. This result is in agreement with Boisgontier et al. (2013), highlighting that older adults are able to manage a postural dual-task as well as younger adults during stable standing conditions. Following Huxhold et al. inverted U-shape model (2006), these secondary tasks were not so challenging to create such cognitive demands to modify mechanisms linking postural control.

### Practical application

The assessment of postural control while subjects are counting backwards aloud is the simplest and most easily-administrable test, with limited instructions than other secondary concomitant demands. Another benefit using the counting backwards aloud test is that secondary task performance is directly noticeable. When measuring dual-task balance, the secondary task is an integral part of the test and real-time assessment should be monitored as well as the simultaneous primary task. A basic example can be explained in asking a patient to make an arithmetic calculation as a secondary task, and later to verbalize the response. When the examiner asks for the solution, at the end of the data sampling, a voluntarily (or not) wrong reply can also be delivered by the subject. Hence, if the subject does not perform any calculation during the assessment, the primary task can result in a biased balance performance (primary task). That would result in an erroneous test result, unlike a genuine result from an incorrect calculation. In this case, it is not possible to verify the authenticity of the reply. When using the Brooks Matrix, the level of error can be more easily quantified, but for this test, the error can be quantified only after the trial.

### Limitations

Although current literature suggests that anthropometric parameters have little influence on balance, some authors (Alonso et al., 2012) affirm that body height should be considered when evaluating static balance posturography and Chiari et al. (2002) suggest to normalize data for height. However, so far, a clear agreement still not exists on this issue yet.

In our study, a significant difference in height between the two groups was present and no normalization of the data was performed. Although this could have potentially affected our results, our sample was randomly selected and the difference in the height of the two age groups is the consequence of the difference in the average height between people born in the forties and in the nineties in Italy.

### Future directions

Recent research emphasizes the importance of calculating dual-task cost to understand the underlying mechanism to improve dual-task performance (Montero-Odasso et al., 2012). We believe that standard methods could be developed to assess balance response under different secondary tasks to formulate a standard approach in a clinical setting. Future studies could focus on large studies to examine the dual-task performance and to retrospectively or prospectively measure falls. Analysis of differing secondary tasks during dual-tasks could then determine which conditions would best predict fall risk.

## Conclusions

The main result of this investigation indicates that a simple verbal assignment was the secondary task which most influenced postural balance. Further, a dual-task condition seems to differently affect balance variables independently from age, inducing an increase (when counting backwards aloud) or a decrease (during arithmetic and visuo-spatial-memory tasks) in the center of pressure velocity and in the sway area. Future research should more fully examine the influence of secondary task choices on fall outcomes to standardize clinical practices to identify individuals who need support in improving dual-task performance.

### Conflict of interest statement

The authors declare that the research was conducted in the absence of any commercial or financial relationships that could be construed as a potential conflict of interest.
