# Supplementary material for: Influence of age on postural sway during different dual-task conditions
Source: Front Aging Neurosci. 2014 Oct 22;6:271. doi: 10.3389/fnagi.2014.00271 (PMC4205805; doi:10.3389/fnagi.2014.00271)
Supplement: Supplementary file 1 [file Table1.DOCX]

**Supplementary Table 1. Scores of cognitive secondary tasks**

Supplementary Table 1 shows all scores concerning the secondary dual-task condition. Results are reported for each trial. About spatial-memory Brooks test (SMBT), scores represents the average correct replies where the maximal individual score was 7. About counting backward aloud test (CBAT) and mental arithmetic task (MAT) errors were reported as sum for each group for each trial. * Mann-Withney *U* test indicated a statistical significant difference between young vs. elderly subjects in trial 1 and 2, only on SMBT.

|  | Young subjects (n= 30) | Elderly subjects (n=30) |
| --- | --- | --- |
| SMBT (trial 1) | 6.82 ± 0.37 * | 4.03 ± 2.59 * |
| SMBT (trial 2) | 6.46 ± 0.97 * | 4.26 ± 2.53 * |
| CBAT (trial 1) | 3 | 5 |
| CBAT (trial 2) | 4 | 2 |
| MAT (trial 1) | 24 | 24 |
| MAT (trial 2) | 22 | 19 |
